# The Microstructure Evolution and Electrochemical Corrosion Behavior of 7A46 Aluminum Alloy in Different Quenching Conditions

**DOI:** 10.3390/ma15020477

**Published:** 2022-01-08

**Authors:** Yaru Liu, Lu Xing, Qing Zeng, Qinglin Pan, Sheng Li, Jun Liang, Haoru Yang

**Affiliations:** 1 School of Traffic & Transportation Engineering, Changsha University of Science and Technology, Changsha 410114, China; jjxie888@163.com (L.X.); zhiqicsu@163.com (S.L.); lj13974553495@163.com (J.L.); yanghaoru2021@163.com (H.Y.); 2 School of Physics & Electronic Science, Changsha University of Science and Technology, Changsha 410114, China; zqzqcsu@163.com; 3 School of Materials Science and Engineering, Central South University, Changsha 410083, China; pql1963@163.com

**Keywords:** 7A46 aluminum alloy, quench sensitivity, microstructure evolution, electrochemical impedance spectroscopy, potentiodynamic polarization

## Abstract

The quenching condition of aluminum alloy can affect the mechanical property and corrosion resistance of the profile. This paper is aimed at the low quench sensitivity of aluminum alloys. Scanning electron microscopy and transmission electron microscopy were used to analyze precipitate behaviors of the 7A46 aluminum alloy under different isothermal cooling conditions and microstructure evolutions of quench-induced precipitations. The effect of the different isothermal time on the corrosion resistance of the alloy, and the relationship between microstructure and corrosion resistance after quenching were revealed through electrochemical impedance spectroscopy and potentiodynamic polarization tests. Results show that corrosion sensitivity of the quenching-aged alloy is much higher than that of the double-aged (DA) alloy, and the corrosion resistance of the quenched alloy decreases firstly and then increases. Due to the high density of the matrix precipitates, the increased content of the impurity element, the discontinuity of the grain boundary precipitates and the widening of the precipitates free zone, the most serious degree of corrosion performance among the quenched alloys is 295 °C at 800 s, and the self-corrosion potential and self-current density is −0.919 V and 2.371 μA/cm^2^, respectively.

## 1. Introduction

As the typical heat-treatable strengthening alloys, the performances including mechanical properties and corrosion resistance of Al-Zn-Mg-(Cu) alloys can be dramatically enhanced by solution treatments, quenching processes and ageing treatments [1]. Quenching needs to proceed with an extremely fast cooling rate to retain more solute atoms and vacancies, which are a prerequisite of further strengthening treatment like artificially aging, so that it can determine the effects of the precipitation strengthening [2]. If the cooling rate during quenching is relatively low, the strength and hardness cannot reach the best condition in the following aging treatment [3]. Conversely, the large quenching rate may cause greater residual stress and grain deformation, which may lead to the deformation and even cracking of the alloy. The quenching processes of Al-Zn-Mg-(Cu) alloys can be mainly divided into end-quenching method, isothermal cooling method, continuous cooling method and computer simulation method [4]. For instance, the end quenching method evaluates the quenching sensitivity of alloys by studying the hardenability of alloys, which is called “end quenching method” for short. The isothermal cooling method refers to that during quenching, where the alloy is isothermal treated at different temperatures and for different times, and the conductivity is measured after water cooling, so that the temperature-time-transformation curve of the alloy can be obtained, namely the TTT curve. Continuous cooling method refers to the quenching treatment of the alloy in the different quenching medium after solution, followed by artificial aging, which can obtain the microstructure and properties of the alloy continuously cooled at different quenching rates. Under normal circumstances, the quenching rate of Al-Zn-Mg-(Cu) alloys should be as fast as possible in order to obtain the highest mechanical properties and corrosion resistance [5]. The quench sensitivity of Al-Zn-Mg-(Cu) alloys is affected by the chemical compositions, grain structures and second phases. Many studies have shown that the Al_3_Zr particles form with the addition of Zr, which were coherent with the Al matrix and could hardly become the nucleation center of the quenched precipitates [6]. During quenching processes, the formation of quenching precipitates can consume a large number of adjacent quenching vacancies and solute atoms, leading to the formation of poor vacancies areas and solute atoms deficient regions. These phenomena lead to the decrease of precipitate density after the later ageing treatments, which dramatically weaken the mechanical properties of the alloys [7]. Li et al. [8] investigated the effect of main element contents on the quench sensitivity of 7085 aluminum alloy and found that more content of the Mg and Cu would enhance the quench sensitivity of the studied alloy. Li et al. [9] revealed that the degree of recrystallization has a significant effect on the improvement of quenching sensitivity of the Al-7.59Zn-1.65Mg-1.54Cu-0.11Zr alloy. Starink et al. [10] constructed a physical model to simulate the constant cooling rate and predicted the quench sensitivity of Al-Zn-Mg-Cu alloy through the model. Deng et al. [11] discussed the effects of Mg on the Al-8.0Zn-(1.0–2.0)Mg-1.6Cu alloys, and indicated that the amount and size of precipitates increased by prolonging the soaking time. However, the number of strengthening phases showed an opposite trend. At the same time, as the quenching precipitate formed, parts of the vacancies at the grain boundaries were also consumed. When the vacancy concentration is lower than the critical concentration of nucleation, the precipitate free zone (PFZ) appears, and the width is broadened. This microstructure characteristic is beneficial to the final corrosion resistance of the Al-Zn-Mg-(Cu) alloys.

For Al-Zn-Mg-(Cu) alloys, electrochemical tests including electrochemical impedance spectroscopy (EIS) and potentiodynamic polarization tests have been widely used to characterize the corrosion resistance and analyze corrosion mechanisms [12,13,14]. The EIS test can provide the information of the corrosion behavior under different corrosion stages, which can analyze the corrosion resistance of the alloy and further determine the development of each corrosion section. Potentiodynamic polarization test is used to express the relationship between electrode potential and polarization current or polarization current density. The steady-state polarization curve can reflect the characteristic between the electrode reaction rate and the electrode potential. Researchers [15] used the potentiodynamic polarization test to characterize the relationship between the Cu content and the corrosion resistance of the AA7004 alloy and found that due to the low content of Cu, only one corrosion breakdown potential existed. On the contrary, there are two corrosion breakdown potentials in the 7xxx series alloys with high Cu content. It is believed that the first breakdown potential is related to the dissolution of small hard particles, while the second breakdown potential is corresponding to the selective intergranular corrosion of the alloy. Birbilis et al. [16] used the potentiodynamic polarization test to measure the effect of Al_7_Cu_2_Fe particles on the local corrosion behavior of 7075-T651 alloy and demonstrated that these particles could maintain the original oxidation-reduction reaction in the observable range. Kong et al. [17] analyzed the polarization curve of the anodic oxide film on the 7475-alloy surface and found that the corrosion potential increased along the positive direction, which dramatically enhanced the corrosion resistance after anodic oxidation. Wang et al. [18] characterized the pitting corrosion behavior of the 7A60 aluminum alloy by EIS tests and revealed that the curve included two high-frequency capacitive reactance arcs and one low-frequency inductive reactance arc during the pitting corrosion induction period.

For the heat-treatable and strengthened aluminum alloy, the heating treatment is an effective way to improve the final property of the alloy. Quenching is one of the heat treatment processes. Unsuitable quenching processes can easily cause alloy strength loss or deformation failure. If the quenching rate is too slow, the strengthening effect of the aging precipitation will be affected, and the alloy will not reach the best mechanical property. While, if the quenching rate is too fast, the internal residual stress of the alloy will increase, leading to the deformation or cracking failure of the alloy. By mastering the quenching sensitivity of the alloy, analyzing the effect of the holding time and the quenching temperature on the structure and properties of the alloy, and knowing the microstructure evolution of quenched precipitates, the subsequent performance of the alloy can be effectively guaranteed. Based on these, this work focuses on the relationship between the microstructure evolution and the electrochemical corrosion resistance of the Al-6.69Zn-1.32Mg-0.22Cu aluminum alloy after quenching. Combined with the previous work with isothermal quenching methods [19], three time points were selected for DA treatment at the nose tip temperature. The quenching-aged and DA samples were simultaneously subjected to electrochemical experiments to detect corrosion behaviors of the alloy under different conditions. For microstructure observation, this study used scanning electron microscopy (SEM) and transmission electron microscopy (TEM) results to analyze the corrosion law and intensity of the studied alloy under the different quenching holding time, which could provide a basis for the further research on the local corrosion process and determine the influence of the quench sensitivity on the corrosion resistance.

## 2. Materials and Methods

### 2.1. Materials and Heat Treatment

The studied alloy was extruded to a sheet by the HaoMei Company (Guangdong, China). The chemical composition was determined to be Zn (6.69 wt.%), Mg (1.32 wt.%), Cu (0.22 wt.%), Zr (0.01 wt.%) and Al (for balance), as shown in Table 1. The alloy was solution treated at 470 °C for 1 h followed by the water quenching. As described in the prior study [20], the alloy was aged to the DA state (120 °C/6 h + 145 °C/10 h) for the corrosion test.

### 2.2. Isothermal Quenching Treatment

Specimens with a size of 15 mm in length × 15 mm in width × 2 mm in thickness were cut for isothermal quenching tests. After the solution treatment, samples were transferred into an isothermal salt bath furnace, kept for a certain time at certain temperature, then water-cooled, and then subjected to DA aging treatment. The transfer time of samples is less than 3 s and the salt bath is a mixed bath of 50% NaNO_3_ and 50% KNO_3_. The temperature of the isothermal salt bath is 295 °C and the holding time is 100 s, 800 s and 1500 s, respectively.

### 2.3. Performance Tests and Microstructure Observations

Hardness tests were carried out on the 401 MVD^TM^ digital micro-Vickers hardness tester (Mitutoyo, Neuss, Germany). The loading load was 500 mN and the dwell time was 15 s. The measurement was repeated 10 times and the average value was calculated as the final value. D60K digital metal conductivity measuring instrument was used to test the electrical conductivity. SEM was conducted on MAIA3 TESCAN, coupling with 20 kV accelerating voltage. Thin foils for TEM were mechanical thinning to 60 μm and punched into 3 mm discs, and then prepared by twin-jet electropolishing in a mixture of 75% methanol and 25% nitric acid at −25 °C. The TEM examination was conducted on Tecnai G^2^ 20, operating at 200 kV.

### 2.4. Electrochemical Tests

Potentiodynamic polarization and EIS tests were conducted on a Zahner Zennium electrochemical workstation (Zahner, Kronach, Germany). A three-electrode system was used to test the electrochemical corrosion resistances of the sample. The sequence of the working electrode (WE), the reference electrode (RE) and the counter electrode (CE) was the tested sample, calomel saturated electrode and platinum electrode, respectively. The tested solution was 3.5 wt.% NaCl solution. The scan range of potentiodynamic polarization curve test was from −1.3 V to −0.6 V and the scan speed was 2 mV s^−1^. The testing frequency range of EIS was from 10^5^ Hz to 0.01 Hz and the AC signal amplitude was 10 mV. The size of the test surface for the potentiodynamic polarization and the EIS test was 10 × 10 mm^2^.

## 3. Results

### 3.1. Hardness and Electrical Conductivity Tests

**Figure 1:**
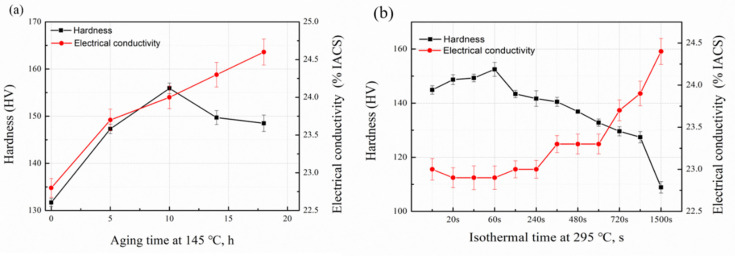
Hardness and electrical conductivity of the sample under DA and 295 °C isothermal conditions: (**a**) DA condition; (**b**) 295 °C isothermal condition.

The result of hardness and electrical conductivity of the solution sample is 48 HV and 35.9% IACS, respectively. The effect of the DA and 295 °C isothermal treatments on hardness and electrical conductivity is shown in Figure 1. As shown in Figure 1a, the hardness value of DA sample first increases and then decreases by prolonging the aging time, which reaches to its peak value (155.9 HV) at 10 h. The electrical conductivity curve shows a monotonous upward trend with the extension of the ageing time. During the 295 °C isothermal treatment, the hardness value decreases obviously and finally reaches to 108.9 HV at 1500 s, as shown in Figure 1b. The electrical conductivity value increases slightly within the first 360 s and then comes to a platform in the later 300 s. Finally, the value reaches 42.1% IACS. It can be seen that the longer quenching holding time can decrease the hardness value while increasing the electrical conductivity value of the alloy. Compared with the two-stage aging alloy, the electrical conductivity and hardness value of the quenched alloy decreases significantly. In general, the higher electrical conductivity value may represent the better stress corrosion resistance (SCC) [21,22]. Therefore, in this work three time points namely 100, 800 and 1500 s were chosen to conduct corrosion experiments in order to compare the electrochemical corrosion resistance with DA sample.

### 3.2. Microstructure Evolution

**Figure 2:**
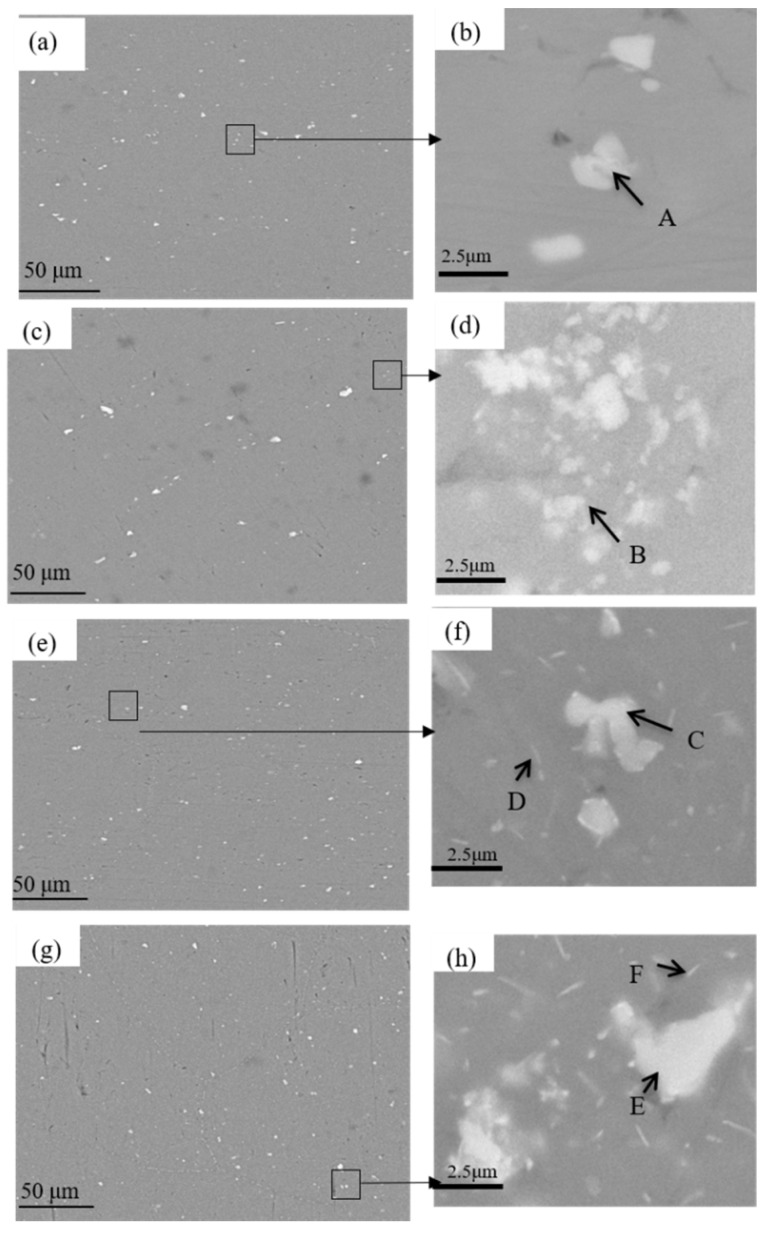
SEM images of the samples under DA and 295 °C isothermal treatments: (**a**,**b**) DA; (**c**,**d**) 100 s; (**e**,**f**) 800 s; (**g**,**h**) 1500 s.

**Figure 3:**
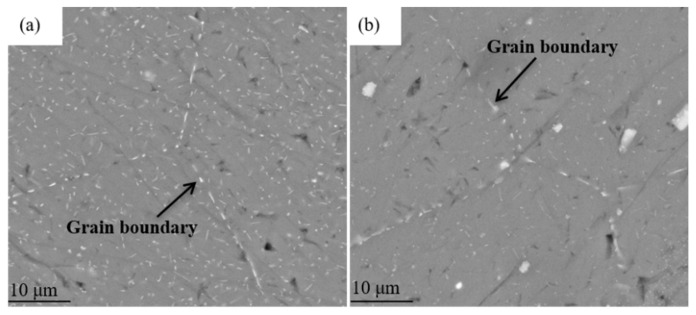
SEM images of the samples quenched at 295 °C isothermal treatment: (**a**) 800 s; (**b**) 1500 s.

Figure 2 shows SEM images of the DA and 295 °C isothermal treatment specimens, the scanned areas of Figure 2b,d,f,h in Figure 2a,c,e,g were boxed out in black line. It can be seen from the Figure 2 that there is a certain amount of second phases precipitated in all tested samples. Most of the coarse phases are rod shaped while the rest is in a needle-like shape. It can be seen in the Figure 2a that most of second phase particles have been redissolved into the Al matrix, and only small residual phases distributed in the DA sample. The low quenching rate has little effect on the residual volume fraction of second phases (Figure 2b). With the increasing of the quenching isothermal time, some dendrite boundaries have been found in the 800 s and 1500 s samples, as shown in the Figure 3a,b. Large quantities of the white quench-induced η phase can be seen. The number of particles in the 800 s samples is obviously more than that in the 1500 s sample, and the quench-induced particles seems a little vague at the 1500 s holding time, due to the small size of the particle. However, the particle on the grain boundaries become obvious when prolonging the holding time, and the length of η phases in 800 s on grain boundaries is shorter than that in 1500 s samples. At the same time, there are a large number of needle-like precipitates, as shown in the Figure 2f,h. These precipitates are white with uneven distribution and different size. The number of quench-induced particles after 800 s isothermal holding time is significantly greater than that of the sample after 1500 s. Grain boundaries are obviously larger and the second phase particles can be seen quite clearly in both 800 s and 1500 s samples. Table 2 illustrates the composition of point A to F phase in each tested samples by Energy Disperse Spectroscopy (EDS) analysis. The Fe element in A point is relatively lower than other three points B, C and E, which indicates that there only a small amount of impurity phases in DA condition. However, the content of Fe in point C is the highest and the atom ratio of Al/(Mg + Zn + Fe) is close to 3:1, which means the residual phase is probably the Al_3_Fe or Al_6_Fe with dissolved Zn and Mg. It can also be observed from Table 2 that a large number of needle-like quench-induced precipitates alloy. The atomic ratios of Zn and Mg in these particles is close to 1:2, representing the η phase (MgZn_2_) precipitate in 800 s and 1500 s samples.

**Figure 4:**
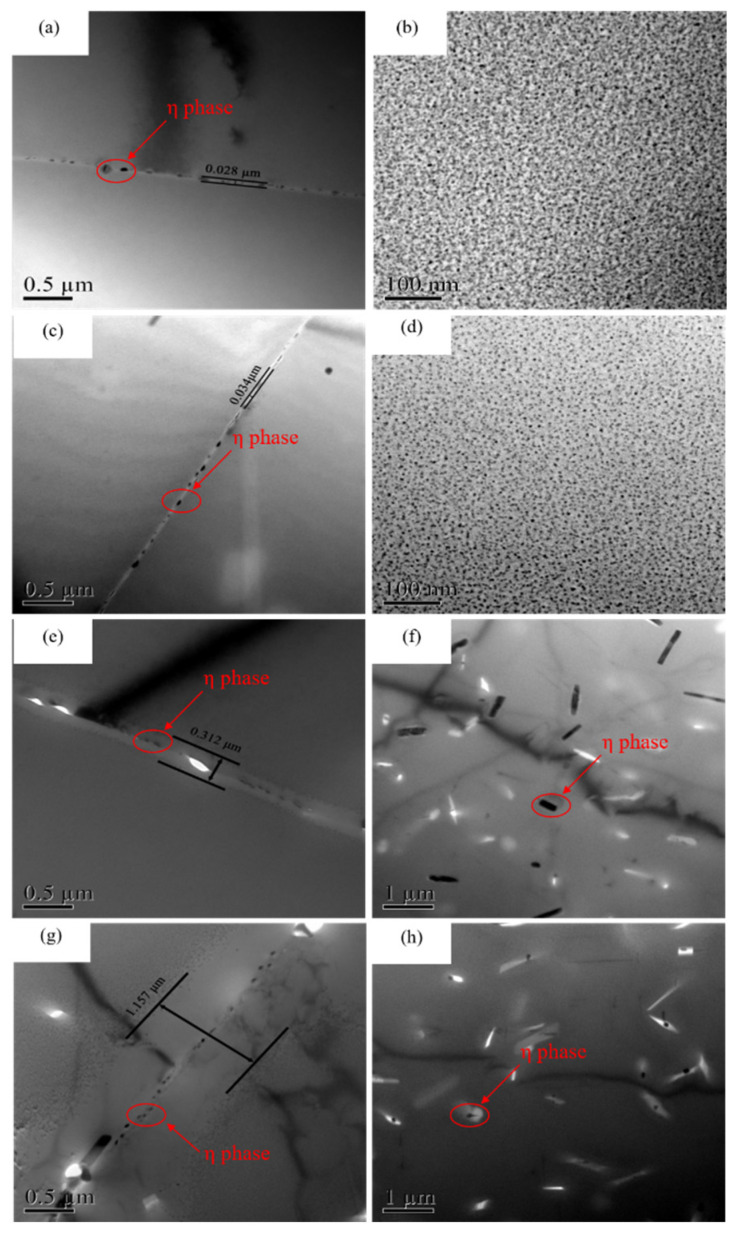
TEM images of DA and 295 °C isothermal quenched samples: (**a**,**b**) DA; (**c**,**d**) 100 s; (**e**,**f**) 800 s; (**g**,**h**) 1500 s.

The microstructure evolution under DA and isothermal treatment is further observed by TEM, as shown in Figure 4. For DA alloy (Figure 4a,b), large-sized spherical particles are distributed in the alloys, and some rod-shaped metastable phases can also be observed. A small number of discontinuously island-liked η phases distribute on grain boundaries. The width of the precipitate free zone (PFZ) in DA condition is 0.028 μm. As the alloy treated at 295 °C for 100 s, the size of precipitates inside grain increases significantly, and the density as well as the volume fraction is lower than that under DA state (Figure 4c). No quenching precipitate was found in this state. Under this state, the distribution of precipitates on the grain boundaries is also discontinuous, but the amount of η phase on grain boundaries is more than that under DA state, as shown in Figure 4d. The size of η phase in 295 °C for 100 s is very close to that under DA state, and the width of PFZ is about 0.034 μm. At the long isothermal holding time, the size and spacing of the η phase increases gradually, as shown in Figure 4e,f. After 295 °C for 800 s isothermal holding time treatment, the coarse second phase can be clearly observed in grain boundaries, as shown in Figure 4e. These particles are discontinuous and distribute as chains. According to Table 2, second phases are MgZn_2_. A clear PFZ can be found near grain boundaries with an average width of 0.312 μm. With the isothermal time increased to 1500 s, many micro-sized particles precipitated on grain boundaries, as shown in Figure 4g. The quench-induced particles can absorb solute atoms around them, leading to the larger spacing between the second phase and the wider of the PFZs (1.157 μm). When the isothermal time is 800 s, despite of the dispersed phase, most of the quenched precipitates are rod-shaped, and a very small part of lath-shaped precipitates are found (Figure 4f). However, under the isothermal time of 1500 s, the size and volume fraction of quenched precipitates obviously reduced, and needle-liked and rod-liked precipitates are staggered in the alloy (Figure 4h).

### 3.3. Polarization Curves

**Figure 5:**
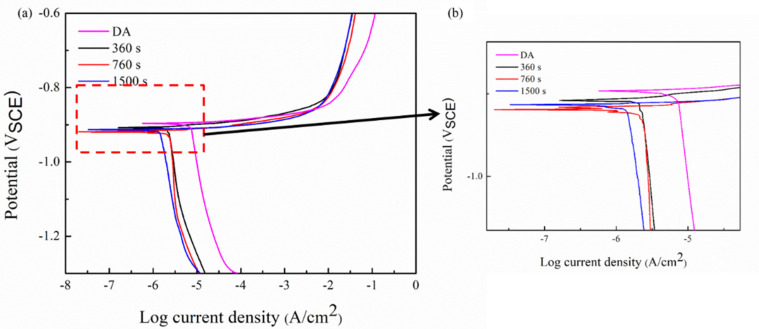
Polarization curves of the alloy under DA and isothermal treatment: (**a**) polarization curves; (**b**) enlarged details.

Figure 5 exhibits the polarization curve in the 3.5 wt.% NaCl solution. The polarization curve measured under DA and different isothermal conditions have a similar trend. The curve in the cathode part is relatively flat, corresponding to the lower electrochemical reaction rate. As the scanning potential approaches the self-corrosion potential, the polarization current decreases gradually and the hydrogen evolution reaction occurs on the surface of polarized samples. When the scanning potential passes through the self-corrosion potential, the polarization current rises abruptly with the positive movement of the scanning potential, proving an active dissolution on the surface. Subsequently, with positive movement of the scanning potential, the increasing trend of the polarization current reduced obviously. The newly formed corrosion oxide layer and the accumulation of corrosion products on the surface have a certain hindrance to the exchange of the electrons between the corrosive solution and the alloy surface, which can reduce the corrosion rate and inhibit the development of corrosion process. Table 3 shows the electrochemical parameters fitted by Corroview software. The self-corrosion potential of the DA sample (−0.896 V) is obviously higher than that of all the 295 °C isothermal treatment samples. The self-current density is also the lowest among all tested samples, which leads to a better corrosion resistance. For the 295 °C isothermal sample, the self-corrosion potential and self-current density of 800 s sample are −0.919 V and 2.371 μA/cm^2^, which corresponds to the highest corrosion sensitivity. The isothermal treatment sample at 100 s and 1500 s have a similar self-current density, which is 1.429 μA/cm^2^ and 1.633 μA/cm^2^, respectively. Results prove that the corrosion resistance decreases firstly and then increases with the increase of quenching holding time.

**Figure 6:**
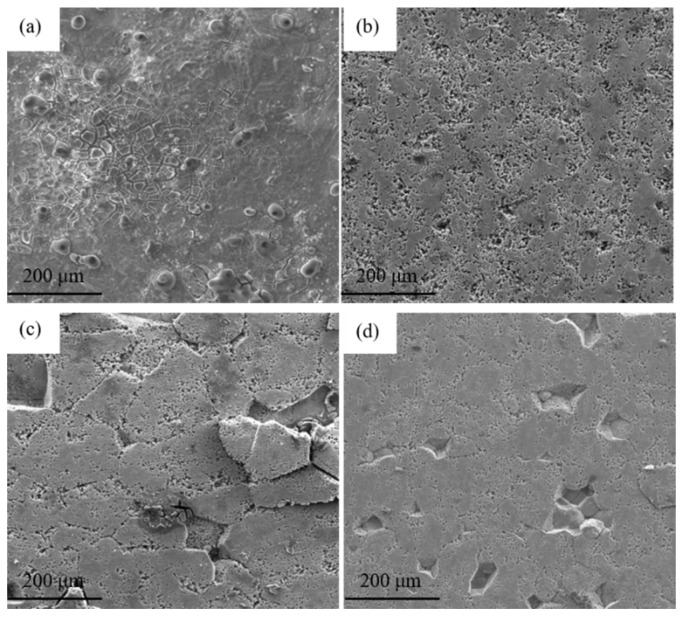
SEM corrosion morphologies of samples after polarization experiments: (**a**) DA; (**b**) 295 °C/100 s; (**c**) 295 °C/800 s; (**d**) 295 °C/1500 s.

SEM images of the surface after polarization curve tests are shown in Figure 6. Corrosion morphologies are obviously different. As shown in Figure 6a, the microscopic surface is relatively smooth, and grain boundaries can be observed, indicating that the corrosion expands from grain boundaries into grains under DA state. At the same time, blister-liked pits appear on the corroded surface, which demonstrates the main corrosion mode in DA sample is pitting corrosion and the anodic dissolution reaction relies on the pitting corrosion. For the 100 s isothermal treatment, the surface is rather frizzy, and the number of pits increases significantly. The corrosion depth increases, and the corrosion process further etches into the Al matrix along the pitting site (Figure 6b). It can be seen from Figure 6c that the surface was cracked and muddy, with some white colloidal corrosion products scattered. Studies have showed that the corrosion products on the surface of Al-Zn-Mg-(Cu) alloy are mainly Al(OH)_3_ [20,23]. Meanwhile, some rough parts of the surface layer dissolved and peeled off as the corrosion continued, and the corrosion degree was extremely serious at 295 °C for 800 s. Compared with this sample, the corrosion degree has alleviated for 1500 s. In addition, it could be found that the alloy has relatively large flakes around the pitting holes, which illustrated that the electrochemical corrosion was carried out along the extension of the corrosion cracks.

### 3.4. EIS Test

**Figure 7:**
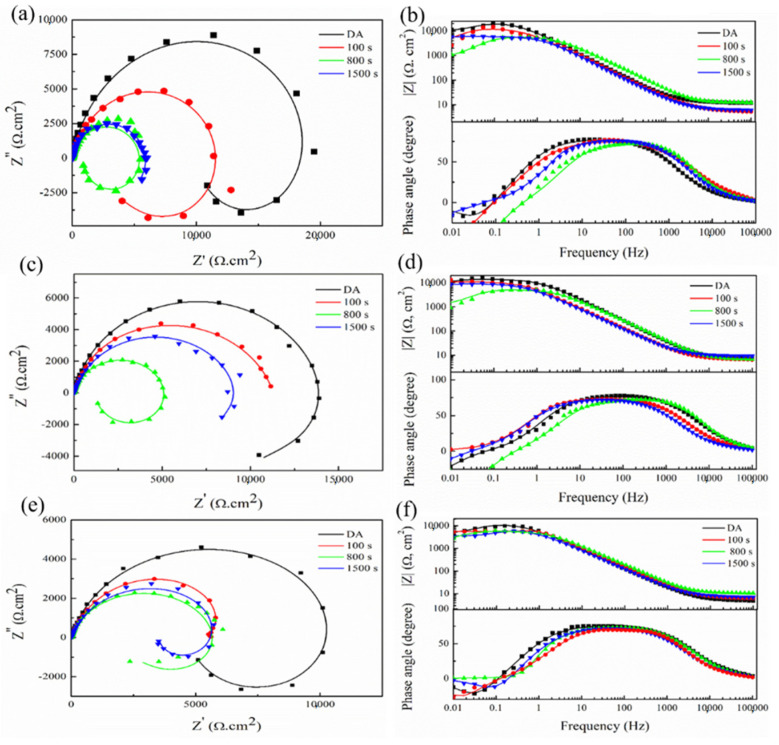
The Nyquist and Bode plots of tested sample under DA state and 295 °C in different isothermal holding time (The point represents the actual measurement results and the solid line stands the fitting results): (**a**,**b**) 0 h; (**c**,**d**) 48 h; (**e**,**f**) 96 h.

**Figure 8:**
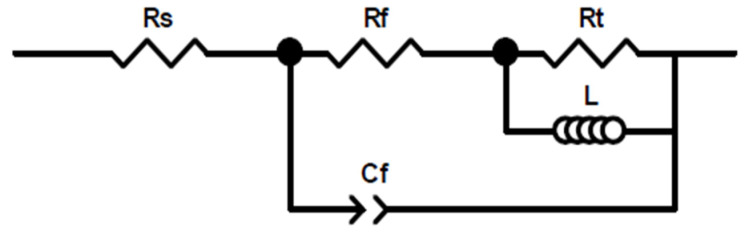
The equivalent circuit diagram: R_s_ represents the solution resistance; C_f_ and R_f_ are the capacitance of the corrosion layer and the resistance of the corrosion product layer, respectively; R_t_ stands the charge transfer resistance and L is the inductance of the adsorbed species.

EIS tests were used to analyze the corrosion mechanism under different conditions [24] and reflect the sensitivity of the studied alloy to pitting and exfoliation. Figure 7 exhibits EIS results of the alloy under different states for different soaking times, including 0 h, 48 h and 96 h. The equivalent circuit [25] of the samples is shown in Figure 8.

The Nyquist image including two parts: the Capacitive Loop (CL) in high frequency and the Inductive Loop (IL) in low frequency. The CL arc is considered to be related to the charge transfer reaction between the electric double layer on the surface of the corrosion medium interface. The IL arc relates to the weakening of the protective effectiveness of the oxide layer during the anodic dissolution of the aluminum alloys [26]. As shown in Figure 7, with prolonging the immersion time, the oxide film is eroded and becomes thinned, exposed to the corrosive medium. This process can be reflected in the Nyquist diagram that shows the continuous reduction of the IL arc and the significant shrinking of the CL arc with the extension of the immersion time, which indicates that the corrosion rate increases. It is worth noting that the alloy under DA state has the largest capacitive arc at the initial stage of the corrosion, while the alloy under 800 s isothermal holding time has the smallest capacitive arc. The large arc represents good corrosion resistance, and the alloy under DA state has the best corrosion resistance at the beginning of the corrosion. It can be seen from Figure 7b,d that only one phase peak appears in the mid-frequency stage (10 Hz to 10^3^ Hz), which means that the corrosion mainly extends downward from the surface layer in the former 48 h immersion [27]. However, with the prolonging of the immersion time, another phase peak is shown in the low-frequency stage (0.01 Hz to 0.1 Hz), as shown in Figure 7f. The appearance of the second peak means that the exposed new alloy surface begins to be corroded, as the original metal surface continues to decrease. At the same time, the phase angle gradually decreases with extending the corrosion time, which also demonstrates the attenuation in the corrosion resistance.

The corresponding fitting values of the tested samples are listed in Table 4. Among them, R_t_ is usually used to indicate the resistance of charge transfer on the metal surface, and the value is inversely proportional to the corrosion reaction rate of the metal [24]. The n_l_ is related to the surface roughness of the electrode. As shown in Table 4, the value of n_l_ decreases in relation to the corrodes of the samples. Furthermore, the value of R_t_ decreases dramatically with prolonging of the immersion time, indicating that the impedance decreases, and that the corrosion rate increases. In the former 48 h, due to the gradual penetration of the corrosive solution, the R_t_ of DA state sample decreases from 1.097 × 10^4^ Ω·cm^−2^ to 8.553 × 10^3^ Ω·cm^−2^. For 295 °C quenched 800 s sample, the value of R_t_ decreases from 4.837 × 10^3^ Ω·cm^−2^ to 3.041 × 10^3^ Ω·cm^−2^. The descend range of R_t_ value is 6.037 × 10^3^ Ω·cm^−2^ and 2.643 × 10^3^ Ω·cm^−2^, under 100 s and 1500 s holding time state, respectively. EIS results prove that the DA sample has better corrosion resistance compared with 295 °C quench samples. Moreover, the sample under 800 s holding time state has the worse corrosion resistance among all the quenched samples.

## 4. Discussion

Experimental results show that the formation of quench-induced precipitates consume a large amount of nearby quenching vacancies and solute atoms during the isothermal treatment, resulting in a lean vacancy area and solute-poor regions around the precipitates, and decreasing the number of atoms used as age-hardening precipitates. As a result, the final mechanical properties after the quench process decrease obviously. With prolonging the isothermal holding time, the percentage of quenched precipitates increases, which increases the final electrical conductivity of the studied alloy. From Figure 1a, we could observe that by prolonging aging time, the hardness curve first increased and reached to the peak value, before it decreased. Combined with Figure 4a,b, this was owed to the size of the precipitates. With the extension of holding time, the size and number of precipitated phases increased obviously, and the amount of strengthened phase decreased, leading to the decrease of the sample’s hardness. While the number of the precipitate increased, the electrical conductivity of the alloy was enhanced. As a result, the electrical conductivity curve in Figure 1a shown a continuous upward trend. While the quenched alloy, the hardness curve and the electrical conductivity curve exhibited the same trend as Figure 1a when the holding time was prolonged. This is because, in the nose temperature, the η phase would reach to its maximum conversion rate, and the gestation time is the shortest. Thus, by prolonging the holding time at 295 °C, the fastest transition rate of the η phase would be reached, but the formation of coarse precipitates would also be inhibited with the extension of the holding time. Therefore, the mechanical properties of the alloy decreased, but at the same time, due to the increasing number in the second phase, the electrical conductivity continued to increase.

Many factors can affect the corrosion resistance of Al-Zn-Mg-(Cu) alloys, including grain size, re-crystallization degree, the density, size and distribution of precipitates, grain boundary precipitates and the width of PFZ. These factors often interact with each other, and the influence mechanisms are more complicated [15,28]. Regarding corrosion mechanisms of Al-Zn-Mg-(Cu) alloys, three main points are proposed: (I) The different corrosion potential between grain boundaries and the Al matrix leading to the occurrence of the galvanic corrosion; (II) The breakdown voltage between the PFZ and the Al matrix is different; (III) The dissolution of the grain boundary precipitate causes the formation of an occlusive erosion environment, leading to a continuous corrosion along the grain boundaries. (IV) The coarse second phases are essential on the occurrence of corrosion.

In this study, the corrosion resistance decreased after quenching processes compared with the DA sample, and the electrochemical corrosion susceptibility decreased first and then increased with the prolonging of the isothermal holding time. This phenomenon is mainly attributed to the differences in amount, size and the spacing of precipitates inside the grains and the grain boundary [29]. Besides, the change of PFZ also has a certain influence on the corrosion resistance of quenched alloys. According to the SEM-EDS results, the existing second phases in the quenched and aged samples, η phase (MgZn_2_) and impurity phases (Al_3_Fe or Al_6_Fe). Compared with Al, Mg is more active and has a more negative electrode potential, and thus the phase is easier to be corroded. In addition, Fe is relatively inactive compared to Al, so that the electrode potential is positive and the formed phase is more difficult to be corroded. When these phases exist in the studied alloy at the same time, the anode phase with more negative potential relative to the Al matrix can be preferentially dissolved. The cathode phase with more positive potential will be protected, resulting in the dissolution of the surrounding Al or anode phases. The potential of η phase on the grain boundaries, PFZ and Al matrix is −1.04 V, −0.57 V and −0.85 V, respectively [20]. The different potential between various phases becomes the driving force for the corrosion. For DA state sample, the discontinuous distribution of the η phase on the grain boundaries and the widened PFZs block the continuity of the electrochemical cell, which inhibits the corrosive medium entering the inside of the Al matrix, improves the corrosion resistance of the alloy. As for the quenched alloys, the size and number of precipitates significantly increased, which enhanced the differences in potential between the Al matrix and precipitates. The activity of electrode pairs is also enhanced, which increases the degree of corrosion. A different holding time has different effects on electrochemical corrosion of quenched alloy. After 295 °C at 100 s isothermal holding time sample, the number of precipitates obviously increases, which accelerates the dissolution of surrounding the Al matrix. The PFZs is only 0.006 μm wider than that under DA state (Figure 4c), which can prohibit the formation of galvanic cells to a certain extent. Therefore, the corrosion performance of quenched alloy after 100 s isothermal holding time is the closest to that of the DA state alloy. For the 1500 s sample, due to the long holding time, the number and volume fraction of precipitates inside grains and grain boundaries continue to increase, as shown in Figure 4g,h. Many solute atoms and vacancies are consumed during the holding time, resulting in the broadening of the PFZs. Song et al. [30] pointed out that when the width of the PFZ exceeded the critical value, it would exhibit cathodic behaviors and many corrosion galvanic cells might formed in grain boundaries, the dissolution process the η phase. Therefore, the corrosion resistance of the alloy decreases at 295 °C at 1500 s holding time. This view convinced that the coarse precipitates can also induce the pitting corrosion [31]. During the corrosion process, blisters are apt to propagate along grain boundaries with coarsened particles [32]. As shown in Figure 2h, the amount of quench-induced precipitates in 800 s sample is the largest among the quenched samples. With extending the corrosion immersion time, the continuous accumulation of corrosion products would produce a wedging force on the vertical corrosion surface of the incompletely corroded grains [20], causing the outer layer to peel off (Figure 6c,d). Meanwhile, as the amount of the coarsened precipitates in 800 s sample is much more than that in 1500 s sample, the potential between the Al matrix and the coarsened precipitates is enhanced in 800 s sample, the dissolution of the Al matrix is accelerated around the coarsened particles. At the same time, it can be seen from Table 2 that for the Fe-containing impurity phases formed in the 800 s sample, the electrode potential is more positive than the Al matrix and promotes the conversion of surrounding O_2_ into OH^−^, which destroys the passivation film on the surface [33]. The linear coarse phases precipitated formed along the grain boundaries would further increase the potential difference (Figure 3a), causing continuous corrosion of grain boundaries. From the electrochemical results, the breaking sequence of passive film surface is 800 s > 1500 s > 100 s > DA state. In summary, although the size of grain boundary precipitates and PFZs increases after being quenched, it increases the corrosion potential as well as the corrosion tendency of the matrix, resulting in low corrosion resistance in all quenched samples. Among the quenched alloy, the 800 s isothermal holding time sample has the worst corrosion resistance.

## 5. Conclusions

In this study, the corrosion resistance and microstructure evolution of 7A46 aluminum alloy, after different quench processes, were analyzed. The following conclusions can be summarized as follows:

1. During the isothermal treatment, the quenched precipitates consume a large number of solute atoms, which decreases the precipitation of age-strengthening phases and results in a decline of the final mechanical properties of the studied alloy.
2. With the extension of isothermal holding time, the size of grain boundary precipitates increases significantly, and the PFZ is widened from 0.034 μm at 100 s to 1.157 μm at 1500 s.
3. The degree of electrochemical corrosion of the quenched alloy increases, showing a quench sensitivity.
4. The corrosion resistance of the quenched alloy increases at first and then decreases with the prolonging of the isothermal holding time. The DA sample has the highest self-corrosion potential (−0.896V) and lowest self-current density (1.017μA/cm^2^), and the 800 s isothermal treatment sample has the lowest corrosion resistance among all the tested samples, at −0.919 V self-corrosion potential and 2.371 μA/cm^2^ self-current density 2.371 μA/cm^2^.

The paper studied the effect of alloy quenching sensitivity on corrosion performance by changing the isothermal time, but lacks the experimental methods to explore the effect of cooling rate on alloy quenching sensitivity. In the next step, different continuous cooling methods can be adopted, such as normal temperature water cooling, mist cooling and liquid nitrogen cooling to study the evolution of the precipitated phase of the alloy at different cooling rates and the effect on properties of the alloy.

## Figures and Tables

**Table 1:** The chemical composition (wt.%) of the studied alloy.

Element	Zn	Mg	Cu	Fe	Si	Mn	Ti	Zr	Al
Composition (wt.%)	6.69	1.32	0.22	0.12	0.04	0.01	0.04	0.01	Bal.

**Table 2:** The chemical composition of second phases in DA and 295 °C isothermal samples (at.%).

Phase	Al	Zn	Mg	Fe
A	93.03	2.79	1.47	2.71
B	90.62	2.18	1.31	5.90
C	84.56	2.49	0.81	12.14
D	92.64	4.61	2.59	0.16
E	89.95	2.98	1.35	5.72
F	93.99	4.08	1.93	0

**Table 3:** Polarization curve parameters of DA and different isothermal time samples.

Condition	E_corr_ (V)	I_corr_ (μA/cm^2^)
DA	−0.896 ± 0.006	1.017
100 s	−0.908 ± 0.012	1.429
800 s	−0.919 ± 0.002	2.371
1500 s	−0.914 ± 0.018	1.633

**Table 4:** Electrochemical parameters obtained from EIS analysis.

Sample State	Time (h)	R_t_ (Ω·cm^−2^)	R_s_ (Ω·cm^−2^)	R_f_ (Ω·cm^−2^)	C_f_ (/F·cm^−2^)	n_l_	L (H)
DA	0	1.097 × 10^4^	12.27	1.021 × 10^4^	3.000 × 10^−5^	8.829 × 10^−1^	3.588 × 10^4^
100 s	0	9.659 × 10^3^	12.04	2.919 × 10^3^	2.158 × 10^−5^	8.490 × 10^−1^	5.735 × 10^4^
800 s	0	4.837 × 10^3^	5.472	8.122 × 10^2^	1.278 × 10^−5^	8.662 × 10^−1^	9.001 × 10^3^
1500 s	0	5.956 × 10^3^	6.277	3.107 × 10^3^	2.685 × 10^−5^	8.831 × 10^−1^	3.229 × 10^5^
DA	48 h	8.554 × 10^3^	7.345	5.601 × 10^3^	1.207 × 10^−5^	8.818 × 10^−1^	1.648 × 10^5^
100 s	48 h	6.740× 10^3^	6.608	1.130 × 10^3^	3.517 × 10^−5^	8.519 × 10^−1^	3.584 × 10^2^
800 s	48 h	4.084 × 10^3^	6.594	5.632 × 10^2^	1.503 × 10^−5^	8.314 × 10^−1^	1.114 × 10^4^
1500 s	48 h	4.431 × 10^3^	9.196	4.911 × 10^3^	3.777 × 10^−5^	8.348 × 10^−1^	1.324 × 10^5^
DA	96 h	6.44 × 10^3^	4.895	4.847 × 10^3^	3.082 × 10^−5^	8.637 × 10^−1^	2.072 × 10^4^
100 s	96 h	3.582 × 10^3^	10.39	2.307 × 10^3^	2.359 × 10^−5^	8.414 × 10^−1^	2.43 × 10^4^
800 s	96 h	3.041 × 10^3^	6.268	1.225 × 10^2^	2.783 × 10^−5^	8.329 × 10^−1^	6.652 × 10^2^
1500 s	96 h	3.293 × 10^3^	7.108	3.536 × 10^3^	3.870 × 10^−5^	8.492 × 10^−1^	3.739 × 10^3^

## Data Availability

Data sharing not applicable.
